# Loneliness and Emotional and Externalizing Problems in Early Adolescence: Moderating and Mediating Effects of Coping Skills

**DOI:** 10.3390/bs15091192

**Published:** 2025-08-31

**Authors:** Sharmila Vaz, Reinie Cordier, Annette Joosten, Mandie Shean, Robert Kane, Melissa H. Black, Karen Martin, Natasha Pearce, Kevin Runions

**Affiliations:** 1 Western Australian Centre for Health and Ageing (WACHA), School of Allied Health, The University of Western Australia, Perth, WA 6009, Australia; sharmila.vaz@murdoch.edu.au; 2 Ngangk Yira Institute for Change, Murdoch University, Perth, WA 6150, Australia; 3 Department of Social Work, Education and Community Well-Being, Northumbria University, Newcastle upon Tyne NE7 7XA, UK; 4 Curtin School of Allied Health, Curtin University, Perth, WA 6102, Australia; 5 Department of Health & Rehabilitation Sciences, Faculty of Health Sciences, University of Cape Town, Cape Town 7700, South Africa; 6 School of Allied Health, Australian Catholic University, Melbourne Campus, Fitzroy, VIC 3065, Australia; annette.joosten@acu.edu.au; 7 College Psychologist, Lake Joondalup Baptist College, Perth, WA 6027, Australia; mandie.shean@ljbc.wa.edu.au; 8 School of Psychology and Speech Pathology, Curtin University, Perth, WA 6012, Australia; r.t.kane@curtin.edu.au; 9 Department of Community and Clinical Health, School of Allied Health, Human Services & Sport, La Trobe University, Melbourne, VIC 3086, Australia; m.black@latrobe.edu.au; 10 Center of Neurodevelopmental Disorders (KIND), Department of Women’s and Children’s Health, Centre for Psychiatry Research, Karolinska Institutet & Region Stockholm, 17177 Stockholm, Sweden; 11 Faculty of Education, University of Tasmania, Sandy Bay Campus, Sandy Bay, TAS 7001, Australia; karen.martin@uwa.edu.au; 12 Telethon Kids Institute, Perth, WA 6009, Australia; natasha.pearce@thekids.org.au (N.P.); krunions@smho-smso.ca (K.R.)

**Keywords:** adolescent, cope, youth, mental health, school transition, social–emotional health, resilience, well-being

## Abstract

Loneliness is an unavoidable facet of human existence. When chronic and intense, adolescent loneliness is associated with maladjustment over time. A prospective multiple-cohort study examined the links between child-reported loneliness and coping skills and parent-rated child mental health in early adolescence (aged 11.9 years at Time 1; 12.9 years at Time 2), with a total of 266 students from 75 primary and 152 secondary schools. Results indicated that (i) boys and girls did not differ in their levels of loneliness; (ii) boys reported poorer coping, fewer emotional problems, and more externalizing problems than girls; (iii) loneliness in primary school predicted increases in emotional and externalizing problems over time; (iv) problem-solving and non-productive coping mediated the associations between loneliness and emotional problems and externalizing problems; and (v) reference to others’ coping moderated the association between loneliness and emotional problems. Findings suggest that loneliness may potentially erode positive coping mechanisms over time. This study emphasizes the importance of understanding and addressing the impact of loneliness on early-adolescent mental health.

## 1. Introduction

### 1.1. Loneliness in Early Adolescence

Although solitude—being alone—is an inescapable facet of human existence, loneliness is a common reaction to perceived solitude. The subjective experience of loneliness is fundamentally contrary to the objective state of solitude or being alone (Asher & Paquette, 2003). Loneliness can be characterized as an aversive affective state arising from the perceived discrepancy between interpersonal relationships one currently has and those one desires (Peplau & Perlman, 1982). It is a cognitive appraisal of personal social relationships not living up to self-imposed expectations (Asher & Paquette, 2003) and is characterized by withdrawn sociability and ineffectiveness in one’s behaviors (Heinrich & Gullone, 2006). As such, loneliness signals that one’s relationships are inadequate, and it is a crucial marker of difficulties in social relationships. Establishing healthy and effective ways to manage loneliness in adolescence is an understudied aspect of life course theory.

Loneliness in school-aged children is increasingly conceptualized as a multifaceted experience, involving emotional, cognitive, and social–relational dimensions (A. M. Eccles & Qualter, 2021; Maes et al., 2019). Qualitative and mixed-methods studies emphasize that school-aged children’s experiences of loneliness are diverse and not uniformly distressing. Many children describe loneliness using metaphors and personal narratives that reflect internal self-attributions, perceived social disconnection, and unmet expectations for belonging (Chipuer, 2004; A. M. Eccles & Qualter, 2021). Importantly, loneliness is not always viewed as a negative or distressing state. In a study involving Australian children aged 9 to 12 years, Chipuer (2004) found that approximately 40% of school-aged children described loneliness without referencing negative emotions, 10% did not associate it with social deficits, and more than 80% did not equate being physically alone with feeling lonely. Those who did and did not associate solitude with loneliness reported similar levels of loneliness, underscoring the complexity and subjectivity of the experience. These findings highlight the importance of employing nuanced, developmentally informed, and culturally sensitive tools to measure loneliness, particularly during middle childhood and early adolescence.

In this study, loneliness was conceptualized across four interrelated domains: emotional loneliness, perceived peer rejection, unmet relational needs, and perceived social competence. This multidimensional operationalization reflects contemporary frameworks that define loneliness as encompassing emotional, cognitive, and interpersonal dimensions (A. M. Eccles & Qualter, 2021).

Loneliness is a common adolescent experience (Qualter et al., 2015). Although estimates vary, some research suggests a prevalence of 70% (Qualter et al., 2015), with a demographic increase in the rates of loneliness in the adolescent population documented between 1991 and 2014 (Madsen et al., 2018). As children move into adolescence, they seek autonomy from their parents, have greater expectations about their social relationships, and seek loyalty, support, intimacy, and the exchange of beliefs, values, and ideologies with friends (Parker et al., 1999; Parkhurst & Hopmeyer, 1999; Steinberg, 2002; Steinberg & Morris, 2001; von Soest et al., 2020). During early adolescence, students typically navigate the transition to secondary school, a shift that entails changes in social hierarchies, peer relationships, and institutional expectations (Qualter et al., 2015; von Soest et al., 2020). This period often involves a marked change in perceived status within the school environment; for example, students transition from being among the oldest and most socially established individuals in primary school to being the youngest and least experienced in secondary school. Such a decline in relative social standing and familiarity may heighten feelings of vulnerability and social disconnection, thereby contributing to elevated levels of loneliness during this transitional phase. Challenges in social skills (e.g., initiating or maintaining conversations) (Lodder et al., 2016), social processing biases (e.g., hypervigilance to social threats) (Qualter et al., 2013), emotion regulation difficulties, unmet relatedness needs, and strained or limited peer relationships, are acknowledged as significant contributors to loneliness during adolescence (Gallardo et al., 2018). Further compounding these issues, adolescence is a *‘neurally sensitive’* period wherein rapid changes in neural networks associated with social behavior may increase the perception of loneliness (Wong et al., 2018). 

### 1.2. Loneliness and Its Relationship with Emotional and Externalizing Problems

Unresolved loneliness in adolescence may pose concerns for future health and well-being (Goosby et al., 2013). Adolescent loneliness can have detrimental impacts during mid-life (von Soest et al., 2020) and health across one’s lifespan (Hawkley & Capitanio, 2015). Loneliness alters several biological, behavioral, and psychological pathways, leading to poorer health outcomes, including an increased risk of anxiety, depression, and suicidality (J. T. Cacioppo et al., 2006; Holt-Lunstad et al., 2015; Park et al., 2020). Notably, individuals reporting chronic loneliness face a 26% higher likelihood of early mortality when compared to socially connected peers (Holt-Lunstad et al., 2015).

Emotional problems, also known as internalizing problems, pertain to difficulties that are internally directed and encompass symptoms such as a depressed mood, generalized anxiety, low self-esteem, and excessive self-criticism (Achenbach & Rescorla, 2014). Loneliness is consistently observed to correlate with internalizing issues and is broadly acknowledged as a contributing factor to the onset and perpetuation of depression during adolescence (Erzen & Çikrikci, 2018).

Loneliness in children and adolescents is associated with low self-esteem, self-criticism, neuroticism, and social withdrawal (Besser et al., 2003; Kiuru et al., 2024; Parker & Asher, 1993). In general, people who report feeling lonely make less positive and more negative judgments of their quality of social interactions, appraise social situations to be less supportive, and are more cautious, distrustful, and conflicted in their behavior (Hawkley et al., 2003). Social withdrawal, peer rejection, and victimization are significantly associated with both loneliness (Asher & Paquette, 2003) and depression (Benatov et al., 2020) in children and adolescents. Moreover, the effects of loneliness on quality of social interactions are evident after controlling for a depressed affect and neuroticism (Hawkley et al., 2003).

Correlation studies report moderate to high associations between loneliness and depression (Boivin et al., 1995; Dunn & Sicouri, 2022; Erzen & Çikrikci, 2018). Longitudinal data further indicate that childhood loneliness mediates a depressive mood and is associated with social withdrawal and negative peer experiences (Boivin et al., 1995). Over time, chronic childhood loneliness predicts depressive symptoms, even after accounting for demographic characteristics, perceived stress, and social support (J. T. Cacioppo et al., 2006; S. Cacioppo et al., 2015; Qualter et al., 2010). 

In contrast, externalizing problems refer to outwardly directed behavioral issues, including aggression, delinquency, impulsivity, and rule-breaking behaviors (Dunn & Sicouri, 2022). These behaviors may elicit negative responses from peers, leading to social rejection, which in turn can intensify feelings of loneliness. The relationship between loneliness and externalizing problems in children and adolescents has been studied mainly from the perspective of peer acceptance (Ladd & Troop-Gordon, 2003). Peer rejection and aggression appear to have a bidirectional relationship whereby externalizing problems may contribute to peer rejection, and peer rejection also predicts externalizing problems (Tseng et al., 2013). 

Meta-analytical evidence suggests proactive and reactive aggression differentially relate to behavioral and affective outcomes (Card & Little, 2006; Polman et al., 2007). Reactive aggression (also referred to as hostile, defensive, emotional, or hot aggression) is characterized by impulsive and reflexive behavior in response to a perceived interpersonal threat. It is viewed as a product of frustration, whereas proactive aggression (also referred to as predatory, instrumental, offensive, controlled, or cold aggression) is viewed as an acquired behavior in response to reward (Vitaro & Brendgen, 2005). Longitudinal work suggests reactive aggression is linked to loneliness (Card & Little, 2006) and positively related to depressive symptoms, with peer rejection mediating the relation between loneliness and depressive symptoms (Morrow et al., 2006). 

### 1.3. The Role of Gender in Adolescent Loneliness

Gender differences in adolescent loneliness are ambiguous. A 2005 meta-analysis exploring gender differences in adolescent loneliness in studies published between 1980 and 2004 found inconsistent results. No significant gender differences were identified in 19 of the 31 included studies, while significant differences were identified in 12 studies, 9 of which reported boys to be lonelier than girls, with a large average unweighted effect size (*e_s_* = 0.58–0.75) (Mahon et al., 2005), suggesting a potential gender effect. However, the authors cautiously acknowledged that such variability could be attributed to methodological inconsistencies, including sampling, measurement, and cultural factors, and called for further examination (Mahon et al., 2005). 

Building on these earlier findings, Maes and colleagues (Maes et al., 2019) explored gender differences in loneliness across the lifespan using robust multilevel meta-analytic approaches to account for statistical dependencies (i.e., multiple effect sizes within one study) and measurement effects (i.e., use of different measures of loneliness across studies). This analysis revealed no strong evidence of systematic gender differences in self-reported loneliness during childhood or adolescence. These findings imply that, on average, boys and girls are more similar than different in their self-reported experiences of loneliness (Maes et al., 2019).

The absence of uniform mean-level differences does not diminish the significance of gender in influencing loneliness trajectories. Recent research indicates that the interpretation, context, and social implications of loneliness may vary according to gender, especially during developmental inflection points such as the transition from primary to secondary school (A. M. Eccles & Qualter, 2021). For example, gendered expectations of peer affiliation, emotional expression, and help-seeking may influence how loneliness is experienced and managed, even if average levels appear similar. In this study, we take a nuanced view by examining potential gender moderation in loneliness trajectories across the school transition—a period marked by changes in peer dynamics, school climate, and identity formation, which may interact differentially with gendered socialization patterns.

### 1.4. Coping Skills and Their Relationship with Loneliness and Emotional and Externalizing Problems in Adolescence 

Coping comprises cognitive and behavioral efforts to manage external and/or internal demands that strain or exceed personal resources (Folkman & Lazarus, 1988; Lazarus & Folkman, 1984). The coping strategies that adolescents adopt have significant implications for their well-being and functioning. The literature commonly distinguishes between productive (or functional) coping and non-productive (or maladaptive) coping styles. Productive or functional coping styles include “solving the problem”, which involves working on a problem while remaining optimistic, and “reference to others” coping, which consists of turning to others for support. In contrast, dysfunctional or non-productive coping involves ignoring the problem, worrying, and wishful thinking (Folkman & Lazarus, 1988; Frydenberg, 2008; Lazarus & Folkman, 1984). 

As children move into adolescence, they are increasingly required to develop a broader and more context-sensitive repertoire of coping strategies to navigate the growing complexity of their social, emotional and cognitive worlds (Besevegis & Galanaki, 2010). This period is characterized by increased independence and reduced parental involvement, necessitating adolescents to manage their emotions and resolve issues more autonomously (Seiffge-Krenke & Klessinger, 2000). The transition from primary to secondary education signifies a pivotal developmental milestone, frequently involving increased academic demands, evolving peer relationships, and enhanced social scrutiny—elements that test adolescents’ ability to cope (Seiffge-Krenke & Klessinger, 2000).

Adolescents’ coping styles can shape how they experience loneliness. Productive strategies—such as seeking social support, employing problem-focused approaches, and embracing transcendent-oriented methods like faith or spirituality—are correlated with a reduction in social loneliness within this age group (Zammuner, 2019). In contrast, avoidance-orientated approaches predict increased vulnerability to loneliness over time (Lodder et al., 2016; Qualter et al., 2015). Adolescents who depend on non-productive coping behaviors, such as social withdrawal, excessive worrying, or self-blame, tend to remain more vulnerable to loneliness in the long term (Inderbitzen-Pisaruk et al., 1992; McWhirter, 1990; Page & Cole, 1991). Although both lonely and non-lonely adolescents may initially use sad–passive strategies (e.g., crying, sleeping, sitting and thinking, doing nothing, overeating, or watching television) (Van-Buskirk & Duke, 1991), non-lonely adolescents are more likely to shift toward active coping over time, whereas lonely adolescents often remain trapped in non-productive cycles (Van-Buskirk & Duke, 1991).

Coping strategies also relate to other psychological outcomes, including depression and externalizing behaviors. The causal role of coping in depression is challenging to specify; however, previous studies have shown that maladaptive coping is associated with adolescent emotional and adjustment problems (de Jonge-Heesen et al., 2021; Hampel & Petermann, 2006). In adolescents who have been bullied, the use of avoidant (Benatov et al., 2020) and emotion-focused coping strategies (Undheim et al., 2016) is associated with increased levels of depression, while seeking social support is associated with reduced depression (Benatov et al., 2020). There are three divergent possibilities as to the nature of any causal relations between coping and depression. *First*, active or problem-focused coping may act as a protective mechanism (buffer) against depression, while avoidance coping could be a possible risk factor (Roohafza et al., 2014). *Second*, it may be that a predisposition to depression is associated with non-productive coping. For example, lonely people have been shown to exhibit attribution error (i.e., a tendency to underestimate the degree to which behavior is externally caused) and, therefore, exhibit less active coping, as they believe they have little control over their experiences or their loneliness is due to an unchangeable personal attribute (Heinrich & Gullone, 2006). *Third*, it is similarly plausible that both coping styles and depression are interrelated and have a cyclical effect on each other (Frydenberg, 2008). This cycle could result from common biological predispositions (Dean & Keshavan, 2017), stressful life events (Jenness et al., 2019), or both. Finally, coping styles and depressive features may both be caused by unmeasured extraneous variables. The relationship between coping and externalizing problems is similarly ambiguous; however, some evidence has suggested that non-productive coping is related to externalizing symptoms, while the opposite is true for productive and reference to others coping styles (Guerra et al., 2017).

Given this developmental context, the current study examines coping not only as an outcome-related construct but also as a potential mediator or moderator in the relationship between school transition, loneliness, and emotional and externalizing problems. This approach aligns with developmental theories that conceptualize adolescence as a critical window for the emergence of self-regulated coping and supports our aim to identify modifiable mechanisms that can inform early intervention.

### 1.5. The Current Study 

The current study examined the associations between loneliness and emotional and externalizing problems over the transition from primary to secondary school using data from 266 adolescents. The moderating and mediating role of coping with the relationship between loneliness and problem behaviors is also explored. The following hypotheses (H) were examined.

**Hypothesis 1** **(H1).** *When transitioning to secondary school, emotional problems and loneliness will increase, and adolescents will resort to fewer problem-solving coping strategies and more emotional and non-productive coping strategies. This hypothesis builds on the literature documenting transitional stressors and declining social status in early high school as drivers of loneliness and psychological distress (Qualter et al., 2015; von Soest et al., 2020).*

**H2.**  *Experiencing loneliness before transitioning to secondary school will predict emotional and externalizing problems after transitioning to secondary school. This reflects longitudinal findings linking loneliness to depression and peer-related difficulties (Boivin et al., 1995; J. T. Cacioppo et al., 2006).*

**H3.**  *Adolescents’ coping strategies will mediate the relationship between loneliness at the end of primary school and emotional health and externalizing problems after transitioning to secondary school.*

- Loneliness will be positively associated with non-productive coping, which, in turn, will be positively associated with both emotional health and externalizing problems;
- Loneliness will be negatively associated with problem-solving coping, which, in turn, will be negatively associated with emotional health and externalizing problems.

This is grounded in evidence suggesting that maladaptive coping may perpetuate the adverse outcomes of loneliness (Frydenberg, 2008; Guerra et al., 2017).

**H4.**  *The relationship between loneliness in primary school and subsequent emotional and externalizing problems at the start of secondary school will be moderated by the coping strategies used by the adolescent, with problem-solving coping weakening the associations between loneliness and later problematic outcomes and emotion-focused coping amplifying the associations of loneliness and later problematic outcomes. This aligns with models of coping as a resilience or risk factor in adolescent psychosocial adjustment (de Jonge-Heesen et al., 2021; Roohafza et al., 2014).*

By positioning our hypotheses within well-established yet inadequately integrated streams of developmental, emotional, and coping literatures, this study enhances comprehension of the mechanisms that connect loneliness and adjustment during a critical developmental period. Furthermore, it has practical significance for the identification of targets for early interventions designed to mitigate loneliness-related morbidity.

## 2. Materials and Methods

### 2.1. Design

A prospective multiple-cohort design with two data collection points explored the links between loneliness, coping, and emotional and externalizing problem behaviors in adolescents as they transitioned from primary to secondary schools in Australia. The current study is part of a more extensive study on the factors associated with students’ academic, social–emotional, and participatory adjustment across the primary–secondary school transition (Vaz, 2010). Details on the study design, recruitment, and data collection have been published (Vaz et al., 2014a, 2014b). 

### 2.2. Recruitment

Several recruitment strategies were used to maximize reach and representativeness (Vaz et al., 2014a, 2014b). Ethical approval was obtained from all three school sectors and the chief governing bodies that managed disability-related services for school-aged children in Western Australia (WA). To elicit interest, 250 registered primary schools were telephoned, and recruitment packages (containing a letter of invitation, poster, and school sector endorsement letter) were mailed to all school principals. 

### 2.3. Sampling and Participants

Students were eligible for inclusion if they were enrolled in the final year of primary school in WA (grades 6 or 7) in the academic years commencing January 2006 or 2007 and due to transition to either middle or secondary school in January 2007 or 2008. The recruitment period extended from 13 March 2006 to 12 March 2008. Inclusion was limited to mainstream schools across the three WA education sectors (government, independent, and Catholic) in the educational districts of metropolitan Perth and other major regional city centers of WA. 

Written informed consent to participate was obtained from school principals, parents, and teachers, and written assent was obtained from students. All participants were made aware that participation was voluntary and that they were free to withdraw from the study at any time without providing a reason or prejudice. When students declined to participate, even with parental consent, they were excluded. The primary study had ethical approval from the Curtin University Health Research Ethics Committee in Western Australia (WA) (HR 194/2005). 

**Figure 1:**
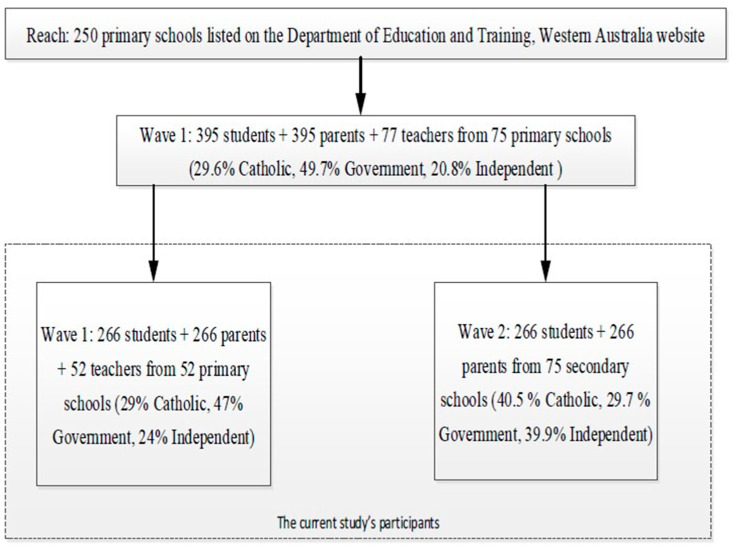
The flow of participants in the study.

Of the 250 primary schools approached, 175 declined, resulting in a non-participation rate of 70% at T1. Questionnaire data were collected from 395 students and their parents from 75 primary schools at Time 1 (T1) and 266 students and their parents from 152 secondary schools at Time 2 (T2), with an attrition rate of 32.7% (Figure 1). 

Data collection intervals were spread over 12 months, with T1 data collected six months before the transition to either middle or secondary school and T2 data collected six months post-transition. T1 data collection commenced in the second semester (Terms 3 and 4) of the final year in primary school (class 6 or 7) in the academic years commencing January 2006 or 2007. The six-month interval provided sufficient time for students to adapt to their new educational environments, with outcomes that could either support or impede their transition. This period coincides with a critical developmental window identified in theoretical and empirical research (J. S. Eccles et al., 1993), making it an appropriate time to establish baseline measures (T1) and assess early post-transition adjustment (T2). By mid-year post-transition (either years 6 or 7), students are typically exposed to substantial changes in school organization, peer relationships, academic demands, and teacher–student interactions (Akos & Galassi, 2004). The current study uses data from 266 students who answered both T1 and T2 questionnaires. 

Chi-square and paired sample *t*-tests demonstrated that the participants who remained engaged in the study at T2 exhibited no significant differences from those who discontinued participation in terms of gender, household SES level, loneliness, coping skills, and mental health functioning scores. This suggests minimal systematic bias among those lost to follow-up.

The mean age of the student sample at T1 was 11.9 years (*SD* = 0.45 years) and at T2 was 12.9 years (*SD* = 0.57 years). Females constituted 53.4% of the sample at T1. There was a shift of enrollment into Catholic and Independent sector schools across the primary–secondary transition, with only 60% of the cohort staying in government schools (Vaz et al., 2014a, 2014b). 

### 2.4. Measurement Tools

Students’ perception of their loneliness in school and coping skills and parents’ perception of their child’s emotional and externalizing problems were examined using the following instruments.

#### 2.4.1. Loneliness in School 

Loneliness in school was assessed using the 16-item Loneliness and Social Dissatisfaction Scale (Asher et al., 1984). The LSDS includes items assessing subjective feelings of loneliness (e.g., “I’m lonely”), peer relationships and social isolation (e.g., “I don’t have any friends”), perceived availability of social support (e.g., “There’s nobody I can go to when I need help”), and social competence (e.g., “I’m good at working with other children”). Students reported the degree to which each statement is a true description of themselves on a five-point scale ranging from 1 (not at all true) to 5 (always true), with reverse ordering for particular items to minimize response set bias (Asher et al., 1984). Previous studies report excellent internal consistencies, with a Cronbach’s α ranging from 0.87 to 0.90 (Asher et al., 1984; Asher & Wheeler, 1985; Goossens & Beyers, 2002). The LSDS has a stable factor structure, is positively correlated with negative peer nominations, and is negatively associated with positive peer nominations and play ratings (Asher & Wheeler, 1985). The LSDS is also reported to have adequate 6-month test–retest reliability (*r* = 0.65, *p* < 0.01) in a middle school sample (Kingery et al., 2011). Higher scores on the LSDS reflect higher perceived loneliness. In the present study, internal consistency was acceptable (α = 0.82), with higher scores indicating greater perceived loneliness.

#### 2.4.2. Emotional and Externalizing Problems

The parent version of the Strengths and Difficulties Questionnaire (SDQ) measured the students’ emotional and externalizing problems (Dickey & Blumberg, 2004; Koskelainen et al., 2001; Riso et al., 2010). Broader externalizing (Conduct Problems and Hyperactivity/Inattention) and internalizing (i.e., Emotional Problems and Peer Problems) subscales are more meaningful for analyses in low-risk samples (Goodman et al., 2010). Nevertheless, to avoid circularity between the LSDS and Peer Problems subscale, only the Emotional Problems subscale of the SDQ was used. Internal consistency of the Emotional Problems and externalizing problem subscales were adequate (*α =* 0.70–0.71) for reliable, independent use in the current study and past research. Weighted mean test–retest coefficients range from 0.66 to 0.76 (Stone et al., 2010). Higher scores reflect more emotional and externalizing problems.

#### 2.4.3. Coping Skills 

The short version of the Adolescent Coping Scale (ACS) measured the usage and helpfulness of 18 coping strategies in general or specific situations (Frydenberg, 1997). In line with evidence that suggests that an individual’s choice of coping strategies is to a large extent consistent, regardless of the nature of the concern (Frydenberg & Lewis, 1994), the general form was used to capture participants’ typical responses to stress. The short version of the ACS also allows for combining scales to produce measures of three empirically defensible coping styles based on factor analysis (Frydenberg, 2008). The ACS classifies these strategies into three empirically derived coping styles, based on factor analytic evidence (Frydenberg, 2008): (i) Productive coping—problem-focused strategies, such as “*seeking to improve oneself*,” “*focusing on solving the problem*,” and “*working hard to achieve.*”; (ii) Productive coping—reference to others, including interpersonal strategies such as “*seeking social support*,” “*seeking professional help*,” or “*investing in close friendships.*”; and (iii) Non-productive coping, comprising maladaptive responses such as “*worry*,” “*tension reduction*,” “*ignoring the problem*,” or “*self-blame.*”. The ACS uses a five-point Likert scale, ranging from 1 (doesn’t apply or don’t do it) to 5 (used a great deal) to rate each item. 

In the Australian context, the ACS was among the most widely used and psychometrically supported tools for youth during the 2005–2010 period, aligning with national research priorities in education and youth well-being (Commonwealth of Australia, 2005; Frydenberg, 2008; Frydenberg & Lewis, 1993; Lewis & Frydenberg, 2004). While loneliness is a distinct psychosocial stressor, loneliness-specific coping measures (e.g., the Coping with Loneliness Questionnaire (Rokach, 2001) had limited applicability or psychometric validation for adolescents in Australian settings at that time. The ACS was therefore deemed the most contextually and developmentally appropriate instrument available to assess general coping profiles relevant to adolescents’ mental health and well-being.

The ACS has established internal-consistency Cronbach’s αs ranging from 0.50 (reference to others coping) to 0.66 (non-productive coping) (Frydenberg & Lewis, 1993). Test–retest reliabilities (14-day intervals) for the same subscales range from 0.44 to 0.84 (Mean *r* = 0.69) on the general form (Frydenberg, 2008). In the current study, the internal consistency of the ACS subscales was adequate (*α =* 0.72–0.80). 

#### 2.4.4. Family Demographics 

Demographic data were collected using items from the Indicators of the Social and Family Functioning Instrument Version-1 (ISAFF) and the Australian Bureau of Statistics surveys (Australian Bureau of Statistics [ABS], 2001). Data on the school postcode, the number of students enrolled in each school, and the organizational structure of each school were obtained from WA Department of Education records.

### 2.5. Analytic Strategy

Data were managed and analyzed using three software packages (SPSS 20.0, M*plus*, Version 5.2, and SAS, Version 9.2). Data from the 2006 and 2007 cohort were alike on all factors. Hence, for purposes of subsequent analyses, sample scores were combined. At scale level, the proportion of missing data ranged from 0.9% to 2.5%. We first assessed the missing data mechanism using Little’s MCAR test (χ^2^, *p* > 0.05), which supported the assumption that the data were missing completely at random (MCAR) (Meyers et al., 2006; Tabachnick & Fidell, 2007). Building on this and in line with recommended best-practice procedures, we used the Expectation–Maximization (EM) algorithm for estimations. For instruments developed or adapted within our broader program of research, missing values were replaced following established scale-specific imputation protocols, as outlined in our previous work (Vaz et al., 2015, 2016). Where no such guidelines existed, data substitution for continuous data was undertaken at an item level before total scores’ computation and replaced using mean scores (Asher et al., 1984; Bourdon et al., 2005; Frydenberg & Lewis, 1993). Sensitivity analysis was conducted to examine the validity of the substitution, which indicated no significant effect on outcomes resulting from the replacement of missing values (McKnight et al., 2007). 

Hierarchical linear modeling (HLM) was used to address potential intra-class correlations resulting from students being nested within schools, which can otherwise inflate Type I error rates and bias standard errors and parameter estimates (Raudenbush & Bryk, 2002). Schools were modeled as Level 2 units to control for between-school variance, thereby providing more accurate estimates of within- and between-student effects. To examine whether clustering at the class and school levels influenced loneliness, coping, and emotional and externalizing problem scores, an HLM was fitted using the MIXED procedure in SAS (Tabachnick & Fidell, 2007). Secondly, gender differences at T1 and T2 were tested to assess whether gender contributed to variations in loneliness, coping styles, and emotional and externalizing behaviors. Thirdly, Pearson correlation coefficients were used to examine associations among variables at each time point and between assessments. Paired sample *t*-tests were then used to compare changes in emotional problems, externalizing problem behaviors, loneliness, and coping skills over time. 

Bivariate correlations among the latent variables were computed to determine the relationship between loneliness and emotional and externalizing problems and, hence, to determine the viability of the mediation analyses. Two mediation models were tested. In both models, loneliness (measured at T1) was the independent variable, and emotional and externalizing problems (measured at T2) were the dependent variables. Both models included problem-solving coping and non-productive coping as mediators. Model 1 measured the mediators at T2, whereas Model 2 measured the mediators at T1. Measurement error was controlled in both models, and standard errors for path coefficients and indirect effects were estimated using a bias-corrected bootstrapping procedure with 1000 resamples in M*plus* (Preacher & Hayes, 2008). This approach is recommended for smaller samples and for indirect effects that may deviate from normality, ensuring more robust and reliable estimates.

Six moderation models were implemented to examine the interactions between coping skills and loneliness in predicting T2 emotional and externalizing problems. Each model evaluated one of the three coping variables at either of the two assessment points. Gender was initially assessed as a potential moderator but was not included in the final models where effects were statistically non-significant, as described in the Results section.

Several criteria were used to measure model fit. Because the raw χ^2^ statistic is influenced by sample size, the χ^2^/degrees of freedom (*df*) ratio was used (Kline, 2005). The Root Mean Square Error of Approximation (*RMSEA*), an index of absolute model fit, was used to estimate how well the model with unknown but optimally chosen parameter estimates would fit the population covariance matrix (Byrne, 1998; Diamantopoulos & Siguaw, 2000). The Non-Normed Fit Index (*NNFI*) [aka Tucker Lewis Index (*TLI*)], an incremental measure of goodness of fit, was used to compare the target model to a more restricted baseline model (Kline, 2005). A good fitting model was considered to have an *NNFI* value of ≥0.95, *RMSEA* < 0.06, and a *CFI* ≥ 0.95 (Hu & Bentler, 1999).

## 3. Results

### 3.1. The Effects of Nested Data on Study Variables Before and After the Transition

At Time 1, the sample comprised 77 classes across 52 unique primary schools. To assess potential clustering effects, we calculated Intraclass Correlation Coefficients (ICCs) for each dependent variable at the class level. The resulting ICCs ranged from 0% to 6%, indicating that the proportion of variance attributable to between-class differences was minimal. This suggests that the hierarchical nesting of students within classes and schools had a negligible impact on the overall variance of student-level outcomes. Given these low ICC values and consistent with established guidelines (Snijders & Bosker, 2012), we determined that the design effect did not warrant multilevel modeling. Thus, for parsimony and interpretability, subsequent analyses, including tests of mediation and moderation, were conducted at the individual student level. 

### 3.2. Descriptive, Correlational, and Inferential Relationships Among Measures 

Correlations between all variables are presented in Table 1. The correlations were 0.20 and 0.21, respectively (both *p* < 0.001). Students’ loneliness scores and coping styles were moderately stable (*r* = 0.50, *p* < 0.001), while emotional (*r* = 0.63, *p* < 0.001) and externalizing scores (*r* = 0.80, *p* < 0.001) were highly stable over the primary–secondary transition period (i.e., across the 12 months). 

Bivariate correlations among the latent variables were computed to determine the viability of mediation analyses. As shown in Table 1, loneliness at T1 showed a low positive relationship with emotional and externalizing problems at T2 (*p* < 0.001). Reference to others coping measured at T1 and T2 were excluded as mediators due to their weak and non-significant correlations with loneliness measured at T1 (both *rs* = 0.0005–0.02, *p* = 0.910). 

Inferential statistics revealed no significant gender differences in self-reported loneliness scores at T1 and T2, supporting the gender similarity hypothesis (Hyde, 2005). Boys, on average, had higher externalizing mental health scores (*M* = 4.78, *SD* = 3.25) than girls (*M* = 3.26, *SD* = 3.20) [*t* (259) = −3.47, *p* < 0.001]. At T2 only, girls were reported to have more emotional problems (*M* = 1.86, *SD* = 1.95) than boys (*M* = 1.38, *SD* = 1.46) [*t* (259) = −2.16, *p* < 0.05]. In terms of coping styles, boys (*M* = 52.02, *SD* = 15.89) were less likely than girls (*M* = 57.31, *SD* = 13.69) to use reference to others as a coping style [*t* (259) = −2.869, *p* < 0.01]. No gender differences were observed in the use of problem-solving coping and non-productive coping. Gender was controlled for in subsequent mediation analyses, given gender differences in emotional problems and externalizing mental health scores.

### 3.3. Hypotheses Testing

The ***first hypothesis (H1)*** was that emotional problems and loneliness would increase, and students would resort less to problem-solving coping strategies and rely more on emotional and non-productive coping strategies when transitioning from primary to secondary school. H1 was partially supported. As outlined in Table 2, emotional problems increased over the transition period (*t* = −2.48, *p* < 0.014), whereas problem-solving coping (*t* = −2.17, *p* < 0.03) and reference to others coping declined (*t* = −2.14, *p* < 0.03). However, there were no significant changes in loneliness scores, externalizing problems, or non-productive coping over time.

***H2*** (pre-transition loneliness would predict emotional and externalizing problems at T2) was supported by linear regression analysis. T1 loneliness prospectively accounted for 4.9% of the variance in T2 emotional problems (Beta = 0.22, *p* < 0.001) and 5.7% of the variance in T2 externalizing problems (Beta = 0.24, *p* < 0.001).

**Figure 2:**
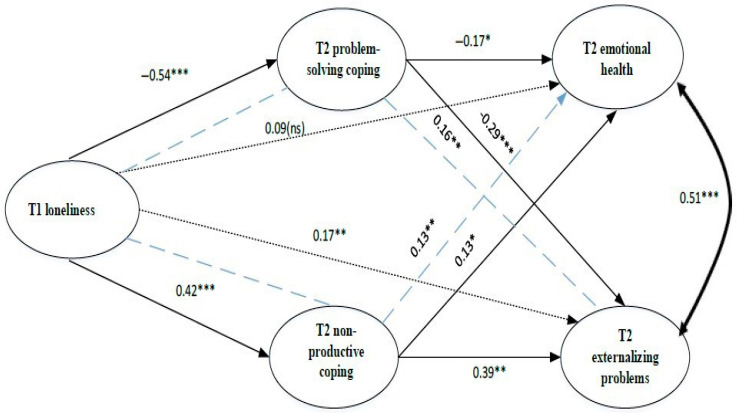
Mediation Model 1(mediators measured at T2). *Note*. * *p* < 0.05; ** *p* < 0.01; *** *p* < 0.001; ns: not significant.

**Figure 3:**
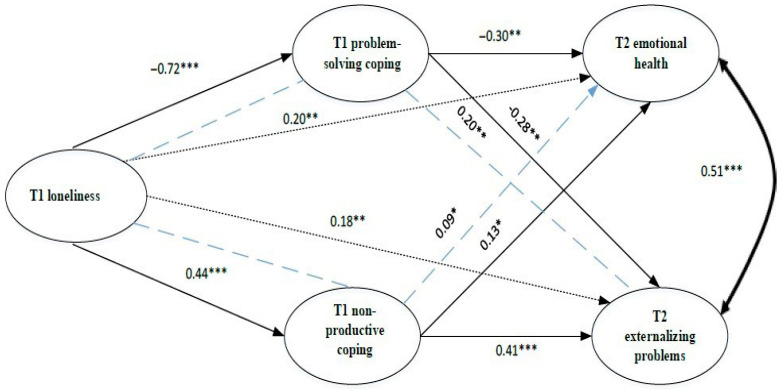
Mediation Model 2 (mediators measured at T1). *Note*. * *p* < 0.05; ** *p* < 0.01; *** *p* < 0.001.

***H3*** was that coping styles would mediate the relationship between loneliness and emotional health and between loneliness and externalizing behaviors during secondary school. H3 was partially supported, as only problem-solving and non-productive coping styles showed evidence of statistical mediation. Three mediating pathways were significant (Figure 2 and Figure 3; Table 3 and Table 4). 

***Model 1***, with mediators assessed at T2, provided a good fit for the data (CFI = 0.995, TLI = 0.984, RMSEA = 0.029 [90% CI = 0.000, 0.113], SRMR = 0.023; Table 3 and Figure 2). Both problem-solving and non-productive coping styles significantly mediated the relationship between T1 loneliness and T2 externalizing problems. However, the mediation was partial, as the direct path from T1 loneliness to T2 externalizing problems remained significant after including the mediators (Figure 2). T2 problem-solving coping styles mediated the relationship of T1 loneliness to T2 emotional health; this was full mediation, with the direct path reduced to non-significance with the mediator included. 

***Model 2***, which assessed mediators at T1 (Figure 3 and Table 4), did not fit the data as well as Model 1 (CFI = 0.945, TLI = 0.817, RMSEA = 0.120 [90% CI = 0.062, 0.186], SRMR = 0.039). In this model, problem-solving and non-productive coping styles were significant in partially mediating T2 externalizing and emotional problems. All four mediation effects were partial mediation, with a direct effect remaining from T1 loneliness to the T2 outcomes in this model. 

**Figure 4:**
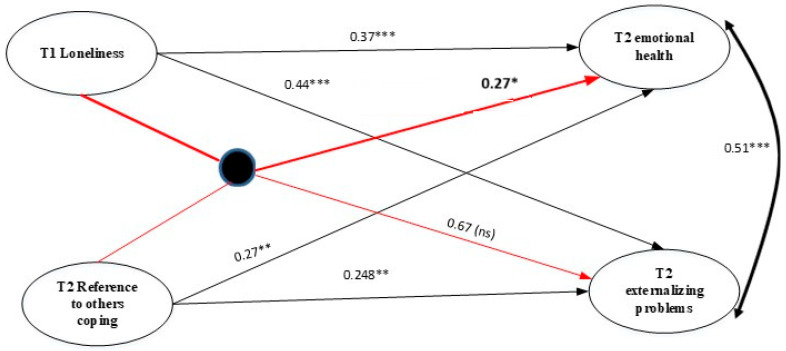
Moderation model (the moderator is RTOC measured at T2). *Note*. * *p* < 0.05; ** *p* < 0.01; *** *p* < 0.001; ns: not significant.

***H4*** was only partially supported, with only one of the six moderation models showing a significant effect (Figure 4 and Table 5). Reference to others coping styles moderated the relationship between T1 loneliness and T2 emotional problems. Specifically, reference to others coping styles amplified the strength of the association between T1 loneliness and T2 emotional health. 

## 4. Discussion

If poorly managed, loneliness during adolescence can contribute to poor health and psychosocial outcomes (von Soest et al., 2020). To our knowledge, this is the first study to demonstrate that coping styles adopted by young adolescents both mediate and moderate their loneliness with subsequent emotional and externalizing problems. 

Significant correlations were found between loneliness in primary school and emotional and externalizing problem behaviors a year later in secondary school. These findings align with extant literature documenting links between loneliness and internalizing symptoms and, to a lesser extent, externalizing problems (Ladd & Troop-Gordon, 2003; Miller-Johnson et al., 2002), providing further support for the necessity of addressing loneliness in young people to prevent adverse psychosocial outcomes (Hawkley & Capitanio, 2015; von Soest et al., 2020).

As predicted, the coping styles employed by adolescents influenced their perception of loneliness, with problem-solving coping styles correlated with lower levels of perceived loneliness and the antipode for non-productive coping. Mediation analysis further indicated that increased loneliness in the last year of primary school predicted more significant use of non-productive coping after transitioning to secondary school (after 12 months), which in turn was associated with increased emotional and externalizing problem behaviors. Conversely, problem-solving coping predicted fewer problem behaviors (both emotional and externalizing). An encouraging finding was that young people with emotional problems also sought help. For example, coping via reference to others, such as seeking support from friends or family, assessed after the transition to secondary school, was associated with emotional and externalizing problem behaviors. The same findings were observed for the associations between coping styles and emotional and externalizing problem behaviors assessed in the last year of primary school before transitioning to high school. These findings are consistent with general trends in our understanding that problem-solving styles are generally adaptive, while emotion-focused coping styles are usually associated with poorer mental health outcomes (Guerra et al., 2017). 

Notwithstanding these findings, it should be acknowledged that the nature of coping styles and what constitutes an “adaptive” or “maladaptive” coping style is nuanced, with the literature suggesting that emotion-focused coping styles can also be adaptive in some instances (Austenfeld & Stanton, 2004). In related literature, Greeff and Van Der Merwe (2004) found that both parents and adolescents indicated family hardiness (family strength and durability), passive appraisal (the tendency to do nothing about the crisis), and social support as coping strategies that helped them to be resilient. Therefore, despite appearing maladaptive, some coping strategies may be functional or beneficial in specific contexts.

Our study’s findings suggest that loneliness before the transition to secondary school may have a lasting influence on students’ concurrent and prospective coping styles, with non-productive coping related to emotional and externalizing problem behaviors. If lonely students develop poor coping styles in primary school, these may be maintained, leading to poor mental health over time (Frydenberg, 2008; Qualter et al., 2010). Therefore, cultivating effective coping strategies may require more deliberate efforts from parents and school staff, as it should not be assumed that students naturally acquire these skills through experience.

In practice, this may entail explicitly instructing individuals in cognitive strategies such as cognitive reappraisal—reframing negative social encounters to diminish rumination—and positive self-talk to bolster self-efficacy (Sheppes et al., 2015; Spyropoulou & Giovazolias, 2024). Behavioral strategies may encompass structured problem-solving processes—such as identifying issues, generating potential solutions, evaluating options, and executing a planned course of action—as well as seeking assistance from trusted adults or peers (Compas et al., 2017; Tang et al., 2021). Social strategies could focus on assertive communication, active listening, and participation in structured peer activities to strengthen a sense of belonging (A. M. Eccles & Qualter, 2021; Lamblin et al., 2017). These recommendations are substantiated by Australian research, demonstrating that the integration of social–emotional learning (SEL), peer support initiatives, and anti-bullying strategies into the school culture—complemented by resources provided to parents for reinforcement at home—can enhance students’ social connectedness, emotional regulation, and resilience during the transition from primary to secondary school (Cross & Lester, 2017; Lester et al., 2013). Whole-school and home–school partnerships of this kind provide a pragmatic, contextually appropriate approach to fostering and maintaining adaptive coping mechanisms during this transitional period of vulnerability (Cross et al., 2018).

Another key finding was that higher loneliness in primary school was associated with less frequent use of productive coping styles and increased externalizing and emotional problems both concurrently and prospectively. As problem-solving coping has been reported to be protective of mental health and well-being, these findings suggest that higher levels of student-reported loneliness may erode the use of problem-solving coping and the protective effects it may have on future mental health. While adaptive coping may indeed result in better mental health outcomes, it appears greater loneliness may be associated with reduced concurrent use of such coping styles. While coping styles were found to mediate the effects of loneliness, problem-solving coping did not appear to shield against emotional problems fully. A caveat, however, is that these results were based on parent reports of externalizing and emotional problems. While the strength of using parents’ perception of students’ health risks is to reduce the risk of shared-source variance in inflating estimates, parental reports may not fully capture adolescents’ perceived experiences or risks. For example, studies have found that adolescents may have experienced more stressful life events that parents perceive as “in the past” but which they are not coping with (Brown & Harris, 1978; Meng et al., 2011). 

Some extant studies illustrate a similar relationship between loneliness and coping styles, as observed in this study. For example, in a cross-sectional sample of college students, loneliness negatively influenced academic adjustment by activating negative coping styles and suppressing positive coping (Quan et al., 2014). Loneliness has also led to depressive symptoms through increased non-productive coping and decreased productive coping using cross-lagged path analyses in a sample of college students (Vanhalst et al., 2012). Such findings suggest that loneliness might reduce the use of productive coping styles and erode any positive effects it may have on mental health. One explanation for this phenomenon is that lonely people tend to exhibit an attribution error, meaning they are more likely to attribute interpersonal issues to personal characteristics and perceive a lower internal locus of control (Heinrich & Gullone, 2006). This may lead them to adopt less productive coping strategies, as they believe their situation is beyond their control. 

The absence of a significant mediation relationship between loneliness and emotional problems over time via problem-solving coping could mean that other protective factors exist, which may need to be considered. For example, emotion regulation coping strategies such as positive reappraisal, being present (savoring), and negative mental time travel (dampening) have been shown to mitigate loneliness (Kearns & Creaven, 2016). Similar to the results observed in our study, peer victimization can also reduce the use of these strategies and lead to greater maladaptive coping, which, in turn, may lead to loneliness (Gardner et al., 2017). Cognitive reappraisal is associated with lower maladaptive coping outside the influence of peer victimization and loneliness (Gardner et al., 2017). Social skills could also be a contributing factor. For example, adolescents may not have the skills to solve problems and, hence, use more avoidant coping strategies (D’zurilla et al., 2004). Other studies have shown that difficulties with social skills can lead to the development of non-productive coping styles, often maintained over time and linked to emotional and externalizing problem behaviors (Compas et al., 2014). This suggests that enhancing social skills during early adolescence could be crucial for fostering more adaptive coping mechanisms.

Social support from one’s peers and friends can reduce the effects of loneliness on students’ mental health (Asher & Paquette, 2003). It is plausible that strong social support could foster more adaptive coping styles. However, despite the importance of social support in prior literature, in the current sample of adolescents, reference to others coping did not decrease levels of loneliness. Interestingly, reference to others coping after transition moderated the relationship between pre-transition loneliness and later emotional problems to the extent that loneliness more strongly predicted emotional problems following the transition to secondary school amongst those who relied more on others for coping. This finding was unexpected, given that seeking social support, including strategies such as talking to friends and seeking advice, is typically considered a functional coping style. The potential influence of personality traits, such as introversion and neuroticism, cannot be dismissed, as these factors were not measured in the current study (Coplan et al., 2021). 

Other studies have demonstrated that seeking social support can reduce depression in young people experiencing peer victimization (Benatov et al., 2020) and can influence loneliness (Zammuner, 2019). Findings from the moderation analysis in our study, however, suggest that lonely adolescents who use reference to others coping styles are likely to have increased emotional problems. Individuals who report feeling lonely may have fewer opportunities to develop social skills. Therefore, while reaching out to others is seen as productive, it may be counterproductive for those with social skill difficulties (Peplau & Perlman, 1982). Alternatively, it may be that students are reaching out to the wrong people—perhaps students are relying on friends from primary school who are no longer at their school or new friends in whom mutual trust has not been fully established, exacerbating loneliness and emotional distress. It may be essential to investigate who is being relied upon to cope more closely and not to presume that all social support is equal. Also, referencing others as a coping strategy is grouped under emotion-focused rather than problem-focused coping in Frydenberg’s classification of coping styles, which is operationalized in the measure used in this study (Lewis & Frydenberg, 2004). While emotion-focused coping can also be adaptive (Austenfeld & Stanton, 2004), it is often viewed as a less effective form of coping, as it deals with the event’s emotions rather than taking steps toward solving the problem. Thus, the challenge that loneliness poses to transitioning to secondary school may be poorly addressed via social support alone, but it can be better addressed through proactive problem-focused coping strategies, such as getting out there and introducing oneself to new students or teachers.

Those not reporting lower levels of reference to others coping may not be making these attempts and receive a perceived or actual rejection from their peers. These considerations may be of particular relevance to young people with known social communication differences, such as autism, a commonly diagnosed neurodevelopmental condition in Australian young people (Australian Institute of Health and Welfare, 2017). Contemporary literature on neurodivergent individuals frames communication difficulties as a *“double empathy problem”*—one arising from a mismatch in communication styles between autistic individuals and their non-autistic peers rather than stemming from the social skills deficits of one individual (Milton, 2012). Such a misalignment may heighten the risk of these children being misunderstood by their peers, rendering reference to others coping ineffectual or even counterproductive when seeking support from peers who may not understand or accept their unique communication styles. 

Moreover, these findings reinforce that loneliness is not just an individual issue but is influenced by the broader social environment. Prejudice, discrimination, and peer victimization can diminish the quality and availability of social support, reducing the effectiveness of reference to others coping strategies. In such contexts, interventions that focus solely on improving individual social skills may be inadequate if environmental stressors are not addressed. Whole of school-based studies in the Australian context have highlighted the importance of whole-school approaches that target peer norms, promote empathy, and foster inclusive climates alongside developing individual skills (Cross & Lester, 2017; Cross et al., 2018; Lester et al., 2013). Integrating these environmental strategies with the development of personal coping skills may assist in ensuring that young individuals—particularly those at increased risk of social exclusion—possess both the requisite skills and supportive environments essential for mitigating loneliness and enhancing mental well-being.

Reference to others coping moderation suggests that loneliness and the cognitive biases that may accompany it erode the possible positive effect of functional coping on mental health. Further research focusing on the nature of those whom the child is referencing and what the nature of the reference provides is required to better model how this coping mode may influence the social and emotional well-being of young adolescents. 

The absence of notable mean-level gender disparities in loneliness within our sample necessitates a more nuanced examination of how socialized gender norms, emotional expression expectations, and vulnerability-related stigma may shape both the experience and reporting of loneliness among adolescents (Maes et al., 2019; von Soest et al., 2020). Evidence indicates that males, owing to dominant masculine norms that discourage emotional disclosure, may exhibit a reduced propensity to identify or report experiences of loneliness. This tendency potentially results in an underestimation within self-report data and increases dependence on externalizing or avoidant coping strategies (Lodder et al., 2016). Conversely, girls might be more sensitive to social and relational cues, possibly interpreting subtle disruptions in peer interactions as indications of exclusion or disconnection.

These gender-specific patterns of emotional processing and social interpretation underscore the limitations of relying exclusively on mean-level comparisons in the examination of loneliness. It is imperative to consider qualitative distinctions in the manifestation of loneliness across different gender groups. In our study, loneliness was measured using the 16-item Loneliness and Social Dissatisfaction Scale (LSDS) (Asher et al., 1984), which captures key emotional and social facets—including perceived peer acceptance, relational support, and social efficacy (Maes et al., 2022). 

Although the LSDS exhibits strong psychometric properties and is extensively employed among school-aged populations, as a self-report instrument, it may be susceptible to gendered response tendencies. Consequently, caution should be exercised when interpreting group-level inferences. These findings highlight the importance of future research incorporating intersectional frameworks to explore how gender interacts with other identity domains, such as race, class, and sexuality, in influencing adolescents’ experiences of loneliness. From a practical standpoint, school-based and mental health interventions ought to integrate gender-sensitive approaches that consider boys’ potential discomfort with emotional disclosure and girls’ vulnerability to relational exclusion. Customizing strategies accordingly may improve both engagement and efficacy of interventions aimed at promoting social connection and emotional well-being during the pivotal transition from primary to secondary school.

Our study’s findings lend support to existing literature regarding loneliness and mental health in adolescents, as well as providing insights into the possible detriment loneliness can have on positive coping. This phenomenon was observed in mediating and moderating pathways through different coping styles. Future longitudinal research could further investigate and establish this potential erosion of positive coping due to loneliness. In this study, we found that positive coping alone may not be enough to protect against mental health issues in adolescents who report being lonely in primary school. A more nuanced understanding of this effect may inform more effective coping interventions, possibly focusing on building strong social skills and networks. Future research may also benefit from additional measurement points to establish longitudinal relationships and their stability. There is a need for further research into the relationships between loneliness, coping, and mental health and their possible associations with social support. Such research can further our understanding of loneliness and what it means for adolescent relationships, coping, and emotional and externalizing problems and inform effective interventions and strategies. 

### Strengths and Limitations

A strength of the current study was the use of validated measures in a large, representative sample of WA schools, with relatively low attrition. The prospective two-wave design facilitated an examination of early psychosocial adjustment during the initial six months of the transition from primary to secondary school—a developmentally pivotal period marked by increased vulnerability. This design yielded valuable insights into short-term changes in loneliness, coping mechanisms, and overall adjustment. Nonetheless, the two-wave framework constrained the ability to model long-term developmental trajectories or determine causality. It is recommended that future longitudinal studies incorporate three or more assessment points to better capture temporal patterns and strengthen the evaluation of mediational pathways and reciprocal relationships over time.

As with all longitudinal designs, this study encountered issues of attrition. This study reported an attrition rate of 32.7%. Consequently, to mitigate the risk of a false positive error, no replacement of missing data was performed. Instead, paired sample t-tests and chi-square analyses were conducted, demonstrating that the participants who remained engaged in the study did not differ significantly in profile from those who withdrew, regarding gender, health status, SES level, and all adjustment outcomes. The results of the paired sample t-tests and chi-square analyses provided a statistical rationale for employing the T1 sample as a reference group in subsequent analyses. Although the missing data were minimal and identified as Missing Completely at Random (MCAR), even low attrition levels can impact the generalizability of findings. Future research may consider incorporating sensitivity analyses (e.g., multiple imputation or pattern mixture modeling) to evaluate potential biases under less restrictive assumptions (Rubin, 2018).

Despite extensive recruitment efforts, caution should be exercised when generalizing this study’s findings, as this study’s cohort was under-represented by the government school sector (Vaz et al., 2014b). This imbalance may restrict external validity, as government schools in Australia disproportionately enroll students from lower socio-economic backgrounds and culturally diverse populations, who often face distinct socio-emotional risks and supports compared with students in non-government schools (Cross et al., 2018; Lamb et al., 2000). 

Further, the present study did not control for socio-economic status or family-related variables, which are known to influence adolescent socio-emotional outcomes (Bronfenbrenner & Morris, 1998; Viner et al., 2012). This omission of family-related variables may have contributed to affecting the observed relationships; therefore, future research should incorporate these contextual factors to better account for potential confounding effects and disentangle them from the psychosocial processes under investigation. Replication of these findings in a population-based study is therefore recommended. Also, the potential influence of caregiver bias in using the SDQ should not be overlooked (Vaz et al., 2016), as parental or guardian perceptions may be shaped by their own mental health, quality of their relationship with the adolescent, or cultural norms around behavior reporting. 

While the current study employed self-report measures to capture adolescents’ emotional and behavioral experiences, it is important to recognize the inherent limitations of this approach. Self-report data are susceptible to several forms of bias, including social desirability, recall inaccuracies, and subjective interpretation of items—factors that may compromise the precision and validity of the findings (Podsakoff et al., 2003). Although anonymity and validated instruments were used to mitigate such risks, these strategies cannot fully eliminate response biases.

An important avenue for future research pertains to the daily strategies utilized by adolescents to cope with loneliness and emotional distress. Qualitative studies reveal that young individuals frequently participate in activities such as watching television, playing video games, or scrolling through social media as methods of distraction (Myruski et al., 2024; Orben et al., 2020). While the Frydenberg and Lewis Adolescent Coping Scale (ACS) encompasses a comprehensive range of coping responses, some contemporary digital-based strategies may be insufficiently represented or classified in ways (e.g., as “avoidance” or “non-productive” coping) that might neglect their potential adaptive functions. Distraction-related activities can offer temporary psychological detachment from distressing stimuli or impart mood-enhancing advantages through participation in enjoyable, familiar routines (Compas et al., 2017). This underscores the necessity to reevaluate the manner in which coping taxonomies, including the ACS, conceptualize such behaviors, especially within digital and media-saturated environments that influence adolescents’ coping repertoires (Coyne et al., 2020).

Although the present study utilized self-report instruments to assess adolescents’ emotional and behavioral experiences, it is essential to acknowledge the inherent limitations associated with this method. Self-report data are vulnerable to various biases, such as social desirability, recall inaccuracies, and subjective interpretation of items—elements that may undermine the accuracy and validity of the results (Podsakoff et al., 2003). Although anonymity and validated instruments were used to mitigate such risks, these strategies cannot fully eliminate response biases.

To overcome these limitations, future research should consider incorporating an Experience Sampling Methodology (ESM), which involves repeated, real-time assessments of participants’ thoughts, feelings, and behaviors in naturalistic settings (Myin-Germeys et al., 2018). ESM reduces reliance on retrospective recollection and advances ecological validity by capturing transient fluctuations in affect and social experiences. This approach is particularly appropriate for understanding the temporal dynamics of loneliness, emotional regulation, and peer interactions—variables that may be prone to distortion in retrospective self-reports. Incorporating ESM could therefore generate more comprehensive, contextually nuanced data that complement and extend the results obtained through conventional self-report methods. ESM has proven to be feasible, reliable, and valid when used with children as young as five years old and across different clinical populations (Cordier et al., 2016; Vilaysack et al., 2016). 

## 5. Conclusions

Loneliness, as a precursor to poor psychosocial and physical health in adolescence, is a growing concern that demands immediate attention. Our study’s findings suggest that loneliness may negatively affect positive coping strategies, highlighting the need for further research and school and family interventions targeting adolescent loneliness and its impact on relationships and coping, as well as emotional and behavioral problems. Encouraging problem-solving coping and reducing non-productive coping may help reduce feelings of loneliness and the emotional and behavioral difficulties associated with it. A partnership between school staff and parents is needed to support young people during this transition period to secondary school. The negative relationship between reference to others coping and loneliness warrants further qualitative scrutiny

## Figures and Tables

**Table 1:** Correlation coefficients for loneliness, emotional problems, externalizing problem behaviors, and coping skills at T1 and T2.

		1	2	3	4	5	6	7	8	9	10	11	12
1	T1 loneliness	1	0.27 **	0.31 **	−0.51 **	0.03	0.36 **	0.50 **	0.21 **	0.21 **	−0.35 **	0.01	0.27 **
2	T1 emotional problems		1	0.47 **	−0.24 **	0.01	0.18 **	0.22 **	0.62 **	0.35 **	−0.09	0.16 **	0.19 **
3	T1 externalizing problems			1	−0.32 **	0.01	0.32 **	0.23 **	0.32 **	0.80 **	−0.24 **	0.07	0.25 **
4	T1 problem-solving coping				1	0.28 **	−0.05	−0.30 **	−0.20 **	−0.23 **	0.45 **	0.04	−0.19 **
5	T1 reference to others coping (RTOC)					1	0.39 **	−0.02	0.02	0.05	0.13 *	0.36 **	0.20 **
6	T1 non-productive coping						1	0.17 **	0.18 **	0.33 **	−0.02	0.22 **	0.45 **
7	T2 loneliness							1	0.24 **	0.23 **	−0.45 **	0.04	0.32 **
8	T2 emotional problems								1	0.36 **	−0.13 *	0.18 **	0.22 **
9	T2 externalizing problems									1	−0.24 **	0.12 *	0.30 **
10	T2 problem-solving coping										1	0.12 *	−0.14 *
11	T2 reference to others coping (RTOC)											1	0.46 **
12	T2 non-productive coping												1
	*M*	29.96	1.89	3.53	71.31	54.83	48.64	30.88	1.64	3.97	69.77	52.62	48
	*SD*	11.15	2	3.12	10.92	14.97	12.52	8.55	1.77	3.31	10.93	14.61	11

Note: T1 = timepoint 1; T2 = timepoint 2; *M* = mean; *SD* = standard deviation. * Correlation is significant at the 0.05 level (2-tailed). ** Correlation is significant at the 0.01 level (2-tailed).

**Table 2:** Changes in loneliness, emotional problems, externalizing problem behaviors, and coping skills over the transition to secondary school (N = 259).

	Paired Differences					*t*	*p*-Value
	*M*	*SD*	*SEM*	*95% CI of Δ Score*			
				*LL*	*UL*		
Δ loneliness	0.92	10.08	0.62	−0.30	2.15	1.47	0.14
Δ emotional problems	−0.25	1.64	0.10	−0.45	−0.05	−2.48	0.01
Δ externalizing problems	0.45	2.01	0.12	0.19	0.69	3.55	0.00
Δ problem-solving coping	−1.53	11.39	0.70	−2.90	−0.14	−2.16	0.03
Δ reference to others coping	−2.22	16.67	1.03	−4.26	−0.17	−2.14	0.03
Δ non-productive coping	−0.64	12.50	0.77	−2.17	0.88	−0.82	0.41

Note. Δ = change; *M* = mean; *SD* = standard deviation; *SEM* = standard error of means; *LL* = lower limits; *UL* = upper limits.

**Table 3:** Standardized path coefficients for Mediation Model 1 (N = 259).

Pathway	Standardized Path Coefficient	*p*-Value (2-Tailed)
T1 loneliness → T2 problem-solving coping	−0.54	<0.001
T2 problem-solving coping → T2 externalizing problems	−0.29	<0.001
T1 loneliness → T2 problem-solving coping → T2 externalizing problems	0.16	0.007
T1 loneliness → T2 non-productive coping	0.42	<0.001
T2 non-productive coping → T2 externalizing problems	0.39	0.004
T1 loneliness → T2 non-productive coping → T2 externalizing problems	0.17	0.002
T2 problem-solving coping → T2 emotional problems	−0.17	0.054
T1 loneliness → T2 problem-solving coping → T2 emotional problems	0.09	0.071
T2 non-productive coping → T2 emotional problems	0.31	0.004
T1 loneliness → T2 non-productive coping → T2 emotional problems	0.13	0.016

T1 = timepoint 1; T2 = timepoint 2.

**Table 4:** Standardized path coefficients for Mediation Model 2 (N = 259).

Pathway	Standardized Path Coefficient	*p*-Value (2-Tailed)
T1 loneliness → T1 problem-solving coping	−0.72	<0.001
T1 problem-solving coping → T2 externalizing problems	−0.28	0.002
**T1 loneliness → T1 problem-solving coping → T2 externalizing problems**	**0.20**	**0.004**
T1 loneliness → T1 non-productive coping	0.44	<0.001
T1 non-productive coping → T2 externalizing problems	0.41	<0.001
**T1 loneliness → T1 non-productive coping → T2 externalizing problems**	**0.18**	**0.001**
T1 problem-solving coping → T2 emotional health	−0.30	0.003
**T1 loneliness → T1 problem-solving coping → T2 emotional problems**	**0.20**	**0.011**
T1 non-productive coping → T2 emotional problems	0.21	0.017
**T1 loneliness → T1 non-productive coping → T2 emotional problems**	**0.09**	**0.035**

T1 = timepoint 1; T2 = timepoint 2.

**Table 5:** Standardized path coefficients for moderation model (N = 259).

Pathway	Standardized Path Coefficient	*p*-Value (2-Tailed)
T1 loneliness → T2 emotional problems	0.37	<0.001
T1 loneliness → T2 externalizing problems	0.44	<0.001
T2 RTOC → T2 emotional problems	0.27	0.002
T2 RTOC → T2 externalizing problems	0.24	0.007
**(T1 loneliness × T2 RTOC) → T2 emotional problems**	**0.27**	**0.033**
(T1 loneliness × T2 RTOC) → T2 externalizing problems	0.67	0.613

*Note*. RTOC = reference to others coping; T1 = timepoint 1; T2 = timepoint 2.

## Data Availability

All relevant data are within the paper.
